# Current and Future Biomarkers for Immune Checkpoint Inhibitors in Head and Neck Squamous Cell Carcinoma

**DOI:** 10.3390/curroncol29060334

**Published:** 2022-06-08

**Authors:** Jong Chul Park, Hari N. Krishnakumar, Srinivas Vinod Saladi

**Affiliations:** 1 Department of Medicine, Massachusetts General Hospital, Harvard Medical School, Boston, MA 02114, USA; Jpark73@mgh.harvard.edu; 2 Long School of Medicine, UT Health, San Antonio, TX 78229, USA; krishnakumah@livemail.uthscsa.edu; 3 Department of Otology and Laryngology, Massachusetts Eye and Ear Infirmary, Harvard Medical School, Boston, MA 02114, USA

**Keywords:** head and neck cancer, immune checkpoint inhibitor, biomarker, PD-L1

## Abstract

With the introduction of immunotherapy, significant improvement has been made in the treatment of head and neck squamous cell carcinoma (HNSCC). However, only a small subset of patients with HNSCC benefit from immunotherapy. The current biomarker, a programmed cell death protein ligand 1 (PD-L1) expression that is widely used in treatment decision making for advanced HNSCC, has only a moderate predictive value. Additionally, PD-L1-based assay has critical inherent limitations due to its highly dynamic nature and lack of standardization. With the advance in molecular techniques and our understanding of biology, more reliable, reproducible, and practical novel biomarkers are being developed. These include but are not limited to neoantigen/mutation characteristics, immune transcriptomes, tumor-infiltrating immune cell composition, cancer epigenomic, proteomics and metabolic characteristics, and plasma-based and organoid assays.

## 1. Introduction

With approximately 890,000 new cases and 450,000 deaths annually, head and neck squamous cell carcinoma (HNSCC) is the 6th most common cancer globally [1]. HNSCCs are malignant cancers that develop along the superficial squamous layer of mucosal epithelium found in the oral cavity, pharynx, larynx, paranasal sinuses, and nasal cavity [1]. Risk factors for HNSCC include the use of tobacco products, alcohol consumption, and viral infections such as human papillomavirus (HPV) and Epstein-Barr virus (EBV) [2]. About 90% of HNSCC presented with a local or locoregional disease, but 10% are diagnosed at an advanced stage with metastatic disease [3]. For local or locoregional diseases, curative surgery and/or radiation therapy are the mainstream therapy [4]. Unfortunately, 50% of patients develop recurrences after the curative-intent treatment, and these recurrences are often not amenable to curative intent salvage therapy and require palliative systemic therapy [5]. 

Over the past decade, there has been a significant advance in systemic therapy for advanced HNSCC with the introduction of immunotherapy [6]. Immunotherapeutic agents that block immune checkpoints, such as programmed cell death protein 1 (PD-1) and cytotoxic T lymphocyte antigen 4 (CTLA-4) have shown promising efficacy with durable antitumor control in a variety of tumors. In HNSCC, immune checkpoint inhibitors (ICIs) that target PD-1, pembrolizumab and nivolumab are currently used in metastatic or recurrent (R/M) HNSCC based on survival benefits [7]. However, only a small subset of patients with advanced HNSCC benefit, with an objective response rate (ORR) ranging from 15–20% and a large proportion of patients suffer from immune-related toxicity from ICI therapy without clinical benefit. As such, identifying reliable and practical predictive biomarkers for optimal patient selection and improved treatment strategy is highly warranted. This review will discuss the current biomarkers data tested in HNSCC and emerging novel biomarkers. 

## 2. Immunotherapy in HNSCC

In HNSCC, immune checkpoint blocking agents pembrolizumab and nivolumab are currently used for the treatment of advanced HNSCC. Both drugs are monoclonal antibodies (mAb) that block PD-1 preventing interaction with PD-L1 and PD-L2 proteins that inhibit T lymphocyte proliferation, and effector functions, and induce apoptosis of tumor-specific T cells [8]. In 2016, both pembrolizumab and nivolumab were approved for the treatment of R/M HNSCC after platinum-based chemotherapy based on KEYNOTE-040 and CHECKMATE-141 studies, respectively, which demonstrated the survival benefit of anti-PD-1 therapy compared to standard chemotherapy [9,10]. Subsequently, in 2019, the FDA granted approval for the use of pembrolizumab as first-line treatment for patients with R/M HNSCC alone or in combination with chemotherapy based on the KEYNOTE-048 study [11]. These ICIs demonstrated durable anti-tumor activity in patients who responded to therapy, with 85% of responses lasting at least 6 months and 71% lasting for over a year [12]. However, the ORR in studied populations was less than 20% [13]. 

## 3. Immune Biomarkers in HNSCC

### 3.1. PD-L1 Expression

Although the introduction of PD-1 inhibitors has increased the utility of immunotherapy for HNSCC, there is still a significant need for more specific biomarkers that can improve the predictive value for ICI response [14]. When considering the general efficacy of anti-PD-1/PD-L1 therapies, it is widely seen that these therapies have a greater response rate in tumors expressing PD-L1 (PD-L1+) than tumors without PD-L1 expression (PD-L1−) across various forms of anti-PD-1/PD-L1 inhibitor therapies and various tumor types [15,16]. The degree of PD-L1 expression within the tumor microenvironment is represented by the following scoring systems: tumor proportion score (TPS), tumor-infiltrating immune cells (IC), and combined positive score (CPS). TPS is the percentage of viable tumor cells that show partial or complete membrane staining for PD-L1 at any intensity. IC is representative of immune cells infiltrating the tumor site, such as T cells, antigen-presenting cells (APCs), and natural killer (NK) cells that show partial or complete membrane staining for PD-L1 at any intensity [17]. CPS is a combination of TPS and IC that analyzes both tumor cells and infiltrating immune cells in the tumor microenvironment that show partial or complete staining for PD-L1 at any intensity [18]. To calculate a CPS, the pathologist must score the number of PD-L1-positive cells (tumor cells, lymphocytes, and macrophages), divide that total by the number of viable tumor cells, and multiply by 100.

### 3.2. PD-L1 Biomarker Data in HNSCC 

PD-L1 expression has been studied and correlated with clinical outcomes across various anti-PD-1/PD-L1 clinical trials (Table 1). Phase 1 KEYNOTE-012 trial studied the efficacy and safety of pembrolizumab in multiple advanced solid tumors including HNSCC. Within this study, a biomarker analysis of PD-L1 expression was conducted by an immunohistochemistry assay (IHC) using the 22C3 antibody. Of 188 patients with HNSCC, both TPS and CPS with a cutoff of 1 were examined for correlation with response to pembrolizumab. Positive CPS (81% of patients) was correlated with higher ORR, progression-free survival (PFS) rates, and overall survival (OS), but such correlation was not observed with positive TPS tumors (65% of patients) indicating a superior predictive value of CPS [19,20]. The study showed that pembrolizumab had a durable anti-tumor effect on responders while exhibiting a manageable safety profile (43), which was the basis for the accelerated approval of pembrolizumab by the food and drug administration (FDA). These results were also corroborated by the phase 2 KEYNOTE-055, which also supported the use of pembrolizumab in patients with pretreated R/M HNSCC [19,21]. Using a 22C3 assay with a CPS cutoff of 1%, ORR was found to be marginally higher among patients with PD-L1+ tumors (18%) when compared to patients with PD-L1− tumors (12%). Additionally, patients with PD-L1+ tumors had slightly higher 6-month PFS (24%; CPS ≥ 1% and 31%; CPS ≥ 50%) compared to PD-L1− patients (20%; CPS < 1% and 20%; CPS < 50%) and comparable rates of OS at 6 months (59%; CPS ≥ 1% and 60%; CPS ≥ 50% vs. 56%; CPS < 1% and 58%; CPS < 50%) [22].

Atezolizumab, a mAb against PD-L1, was tested in patients with advanced HNSCC. In a phase I study, 32 R/M HNSCC patients were treated with atezolizumab and 22% of them achieved objective responses [33]. In a subgroup analysis based on the PD-L1 expression in the tumor-infiltrating immune cell (IC), the PD-L1 + group (n = 25) defined by IC2/3 (≥5%) by the SP142 Ventana assay had a higher ORR (24%) than PD-L1 IC0/1 (<5%) cohort (ORR 14%, n = 7).

The randomized phase III KEYNOTE-040 further studied the efficacy of pembrolizumab among patients with R/M HNSCC who had already undergone platinum-based chemotherapy, comparing it to standard systemic therapy with the OS as a primary endpoint [23,24]. Pembrolizumab demonstrated superior survival compared to the control arm in this population (hazard ratio (HR) 0.80, *p* = 0.016). Although this study substantiated prior trends by showing increased rates of survival in PD-L1+ patients, the PD-L1− based biomarker analysis results of this study were different from those of prior studies; the magnitude of survival benefit of pembrolizumab compared to standard therapy was greater in patients with TPS > 50% (HR 0.53) than patients with CPS > 1 (HR 0.74) [25]. Furthermore, a PFS in patients with CPS > 1 was not different compared to the control group while patients with TPS > 50 had a longer PFS than the control group. Nevertheless, when compared to standard therapies, patients who received pembrolizumab had a greater median OS (mOS) regardless of PD-L1 status and the magnitude of survival benefit was even greater in patients with CPS > 1 and TPS > 50% [11]. 

The CHECKMATE-141 was a phase III randomized study that examined the efficacy of nivolumab as a post-platinum therapy in patients with R/M HNSCC [26]. This study distinguished PD-L1 positivity by tumor expression (TC) ≥ 1% using the 28–8 pharmDx assay. Nivolumab demonstrated a significant increase in OS compared to the standard therapy (HR 0.70, *p* = 0.01) irrespective of PD-L1 tumor expression or HPV status, with estimated 24-month OS nearly tripling from 6.0% to 16.9% for patients on nivolumab; however, the survival benefit was greater with nivolumab in the PD-L1+ subgroup, 57.3% of all PD-L1 evaluable patients, (HR 0.55) compared to PD-L1− population (HR 0.89) [26]. Notably, the magnitudes of survival benefits in subgroups of patients with tumor PD-L1 levels of ≥5% or ≥10% were similar to that with tumor PD-L1 level ≥ 1% with HR of 0.50 and 0.57, respectively.

KEYNOTE-048 study evaluated pembrolizumab as first-line therapy in R/M HNSCC, examining and comparing outcomes of pembrolizumab alone and in combination with platinum-based chemotherapy to the conventional platinum-based combination chemotherapy and cetuximab (Extreme regimen) using co-primary endpoints of OS and PFS [34]. This study used CPS by the 22C3 assay as the PD-L1 expression measure using 2 cut-off values of 1% and 20% [27]. The results of the study supported the efficacy and safety of pembrolizumab alone or in combination with chemotherapy as an appropriate first-line treatment for R/M HNSCC [28,29]. Treatment with pembrolizumab was non-inferior but did not meet the threshold for superiority in survival compared to the extreme regimen in the total population (HR 0.83, *p* = 0.0199). However, pembrolizumab alone demonstrated superior survival in tumors with CPS ≥ 20 (HR 0.61, *p* = 0.0007) and CPS ≥ 1 (HR 0.78, *p* = 0.0086). On the other hand, pembrolizumab with chemotherapy improved overall survival compared to the extreme regimen in the overall population (HR 0.77, *p* = 0.0034). Again, the survival benefits were greater with higher PD-L1 expression; HR 0.60 in CPS ≥ 20 (*p* = 0.0004) and HR 0.65 in CPS ≥ 1 (*p* < 0.0001).

Durvalumab is a mAb against PD-L1, which is currently used for the treatment of lung cancers. In a phase I/II study evaluating durvalumab single agents in multiple solid tumors, an ORR of 6.5% was seen in the HNSCC cohort (n = 62). This study defined the PD-L1 positive population as PD-L1 expression in ≥25% tumor cells (TC) by SP263 Ventana assay. Durvalumab achieved superior objective response and survival in patients with PD-L1+ HNSCC (ORR = 15%; mOS = 8.4 months) than in patients with PD-L1− tumors (ORR = 2.6%; mOS = 7.4 months) [35]. The subsequent phase II study (HAWK) evaluated durvalumab monotherapy in patients with PD-L1-high R/M HNSCC defined as TC ≥ 25% and demonstrated an ORR of 16.2% and a 12-month survival rate of 33.6% [30]. On the other hand, the CONDOR study evaluated durvalumab along with an anti-CTLA-4 mAb tremelimumab, or durvalumab and tremelimumab combination in patients with PD-L1-low/negative R/M HNSCC (TC < 25%). ORR of the durvalumab monotherapy arm (n = 67) was 9.2%. ORR for patients with TC < 1% and TC < 10% were 8.8% and 8.9%, respectively [31]. 

The following randomized phase III study (EAGLE) tested durvalumab vs. durvalumab plus tremelimumab vs. standard chemotherapy in patients with advanced HNSCC regardless of baseline PD-L1 status [32]. In the total population (n = 240), durvalumab monotherapy achieved an ORR of 17.9% and a mOS of 7.6 months, which was not significantly different from the standard control arm (HR 1.04, *p* = 0.76). Median OS in patients with PD-L1 TC ≥ 25% (n = 68) and TC < 25% (n = 172) were 9.8 months and 7.6 months, respectively. 

### 3.3. Challenges and Limitations of PD-L1 as a Predictive Marker

While PD-L1 expression is widely used as a predictive marker to immune checkpoint therapy and is required in certain indications, several challenges and limitations exist. First, several assays utilizing different antibodies for PD-L1 detection are being used and these tests do not always reproduce the same value. When analyzing the concordance between various PD-L1 assays in the HNSCC population, substantial differences in the level of positivity between assays were observed [36]. With percentages of positive immune and tumor cells varying greatly between assays, the eligibility for certain checkpoint inhibitor regimens was dependent on the choice of the assay [37]. Compounded with the fact that there are varying cut-offs determining PD-L1 positivity (>1% and >50%) and a plethora of PD-L1 detection IHC antibodies utilized with unknown comparative performance characteristics, the inconsistencies of these components complicate PD-L1 testing [38]. This begs consideration for other techniques that can more effectively couple the studies of multiple immune markers. In the setting of non-small cell lung cancer, a multicenter study noted there to be strong concordance between 3 of 4 tested assays (28-8, 22C3, and SP263) when scoring tumor cells [39,40,41,42,43]. Although these assays had significantly less concordance when scoring immune cells, it shows promise for future studies identifying more accurate assays that are able to establish a more standardized diagnosis, especially among HNSCC. 

Secondly, PD-L1 expression is highly dynamic and can change significantly over time and by intervening therapies [44]. The data on the effect of chemotherapy on PD-L1 expression is mixed. In NSCLC, while some studies showed that chemotherapy given prior to surgery was shown to increase PD-L1 expression, other studies demonstrated the opposite effect of chemotherapy on PD-L1 expression [45,46]. Additionally, chemotherapy may have distinct effects on tumor cells and immune cells, and different chemotherapeutic agents may have different effects [47]. In HNSCC, Oak et al. demonstrated the cisplatin-based chemotherapy upregulated tumor PD-L1 expression. The data on the effects of the immune checkpoint therapies on the PD-L1 expression is lacking. In an autopsy study, a decrease in PD-L1 expression was observed after pembrolizumab treatment in patients with NSCLC [48].

The current guideline does not specify the timing of the tissue to be tested for PD-L1 or take into consideration any intervening systemic or radiation therapy. This may also potentially explain why some PD-L1-negative patients still benefit from therapy or vice versa, as the immune composition of their tumor microenvironment may have fluctuated since the time of biopsy and the location of the tissue acquisition. Furthermore, it is important to note that HNSCC forms dynamic tumors manifesting high levels of inter- and intra-tumor heterogeneity that has led to significant disparities in therapeutic response to the same treatments [49]. Intra-tumor heterogeneity and intertumoral heterogeneity between primary tumors and nodal metastases were prevalent as well, being seen in 53% of cases [50]. The molecular diversity and continued mutations of HNSCC tumors also make it difficult to obtain truly representative tissue biopsies [51]. Specifically, it can be difficult to determine variations prior to and after medical interventions, as well as during surgical inventions in which it is imperative to identify the tumor nest, stroma, and margin of invasion [52]. 

There is also a lack of standardization based on the location of the biopsy, whether it is a primary, nodal, or distant metastatic lesion. Across several cancers, PD-L1 expression varies across primary and metastatic lesions [53]. This suggests a difference in the immune microenvironment across sites of metastases; however, there are no clear guidelines regarding PD-L1 positivity depending on tissue biopsied. Standardizing the timing and anatomic location of PD-L1 testing can help to remedy many of the discrepancies that exist with immune assays of biopsied HNSCC tissues. In addition, it’s important to biopsy larger samples of tissue, as up to 35% of small volume biopsies were misclassified resulting in false-negative and false-positive results [54].

## 4. Other Biomarkers in HNSCC

The tumor mutational burden (TMB), defined as the total number of mutations found in cancer cells, has been considered as a potential biomarker in assessing ICI therapy [55]. Generally, an increased TMB results in the production and expression of more neo-antigens on MHC proteins that can be recognized by T cells [56]. In a retrospective study analyzing 12 clinical trials including over 1770 patients with solid tumors (including 235 HNSCC patients) treated with pembrolizumab, a high mutational burden (with a cut-off value of 175 mutations/exome) was associated with improved ORR (*p* = 0.016), PFS (*p* < 0.005), and OS (*p* = 0.029), independent of PD-L1 expression [57]. 

Similarly, the quantity of neoantigen has been explored for its predictive value for ICI response. Cancer neoantigens are aberrant antigens produced by cancer-specific genetic alterations such as mutation, alternative splicing, and gene arrangement as well as tumor-specific viral genes. Immunogenic neoantigens are determined based on the affinity of putative neoantigens for the patient-specific HLA class I molecules using prediction algorithms. High neoantigen load has been associated with a high level of tumor-infiltrating immune cells and high expression of pro-immune gene expression in various solid tumors [58,59]. In melanoma patients, neoantigen load was found to be significantly associated with the clinical benefit of ipilimumab [60]. Such predictive value of neoantigen load has been reported in other tumor types including urothelial carcinoma, renal cell carcinoma, and lung cancer [61,62,63,64]. In HNSCC, in addition to single nucleotide variation-derived neoantigens, a high level of frameshift insertion-and deletion-derived neoantigens and HPV/EBV viral antigens may generate anti-tumor immune responses [65]. Hanna et al. reported a higher frequency of frameshift events in ICI responders compared to non-responders in patients with HNSCC [66]. Immune cell infiltration into the tumor microenvironment has also shown prognostic implications [67,68]. Analysis of the levels of CD4+ helper T cells, CD8+ cytotoxic T cells, and FoxP3+ regulatory T cells demonstrated an association of higher tumor-infiltrating lymphocytes (TILs) with improved OS and disease-specific survival even after controlling for other variables. Furthermore, CD4+ cell-rich tumors were better candidates for treatment and associated with higher OS rates than CD4+ cell-depleted tumors [69]. It was also shown that there was a positive correlation between CD3+ T cell infiltration and favorable clinical outcomes in the treatment of HNSCC tumors [70]. Recent studies unveiled the role of tissue-resident memory CD8+T (Trm) cells in anti-tumor immunity. These CD103+CD8+ Trm cells play roles in protective immunity. In human cancers, tumor-infiltration Trm cells have been found to promote anti-tumor immunity and are associated with improved survival in HNSCC [71,72]. Trm cells in tumors are highly enriched with immune checkpoints including PD-L1 and LAG3 and are expanded significantly early with ICI therapy and the levels of CD8+CD103+ T_RM_ are associated with improved patient survival suggesting a critical role Trm in ICI-mediated immune response and its potential predictive value [71,73]. In addition to tumor-infiltrating immune cell composition, the expression of specific immune-related genes in TME has been shown to correlate with clinical outcomes with ICIs in the HNSCC population. Analysis of IFN-γ gene signature of 18 genes (T-cell-inflamed gene expression profile, Tcell_inf_GEP) in HNSCC patient tissues from KEYNOTE-012 study showed independent predictive values of TMB and GEP score with ORR to pembrolizumab therapy response in HPV and EBV negative HNSCC. Interestingly, only the GEP score showed a correlation with pembrolizumab response in HPV- or EBV-mediated tumors [74]. A single-institution retrospective analysis of genomic and transcriptomic data of patients with HNSCC treated with anti-PD-1/PD-L1 ICI showed a correlation between somatic frameshift events and objective responses (*p* = 0.03) [75]. 

As the tissue-based biomarker is not always feasible and as in PD-L1 expression there is significant inherent heterogeneity in tumor tissue, blood-based biomarkers have been explored as an alternative predictive marker to tissue-based ones. Baseline and on-treatment dynamics of peripheral immune cell numbers and/or ratios were examined for potential predictive values. A greater pre-treatment neutrophil-lymphocyte ratio (NLR) is associated with poorer overall rates of survival [76]. A meta-analysis of 6479 patients further corroborates this as combined HR for OS in patients with elevated NLR was 1.78 (95% CI 1.53–2.07; *p* < 0.001) relative to patients with low to normal NLR [77]. NLR has also been shown to exhibit a predictive value for anti-PD-1 therapy response, as low NLR (<6.2) 6 weeks into anti-PD-1 therapy was associated with longer PFS (8.7 vs. 2.9 months, *p* = 0.001) [77]. Circulating tumor DNA-based TMB (bTMB) was evaluated as a predictive marker for immunotherapy in advanced HNSCC. In the randomized phase 3 EAGLE study where R/M HNSCC patients received chemotherapy, durvalumab, or durvalumab and tremelimumab, a higher bTMB was associated with a greater survival benefit of ICIs over chemotherapy [78].

## 5. Novel Biomarkers and Future Directions

**Figure 1:**
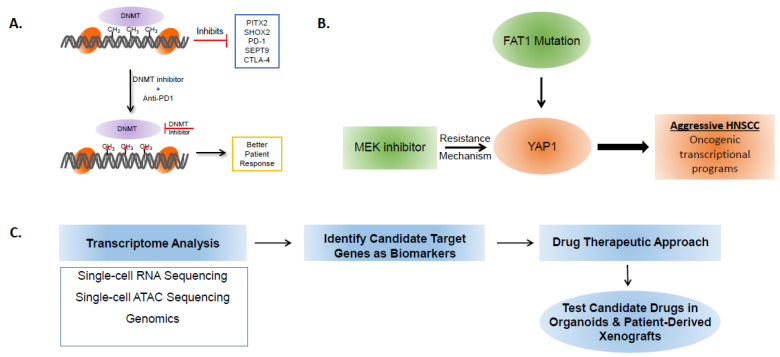
Biomarkers and strategies to target oncogenic programs in HNSCC. (**A**) DNA methylation transferases (DNMT) promote DNA methylation and suppress the expression of immune programs. This could be reactivated through utilization of DNMT inhibitors (DNMTi). DNMTi when combined with anti-PD1 therapy could elicit better response in patients and improve survival. (**B**) Trametinib (MEK inhibitor) resistance and FAT1 mutation results in increased YAP1 mediated transcriptional programs. This could result in aggressive disease state in HNSCC. (**C**) Strategies utilizing single cell technologies and genomics can be utilized to address tumor heterogeneity and also identify novel transcriptional programs or biomarkers. This could help in development of novel therapeutic opportunities. The new drugs can be screened in patient-derived organoids and xenografts for their efficacy and ability to control tumor growth in HNSCC.

There are several emerging techniques being developed and utilized in order to identify reliable, reproducible, and clinically relevant biomarkers for early diagnosis, detection, and development of novel therapeutic strategies (Figure 1). Currently, there are limited biomarkers and few targeted approaches for the treatment of advanced HNSCC [79]. There is a need to utilize recent advances including single-cell sequencing approaches, spatial transcriptomics, epigenetic technologies including single-cell ATAC-sequencing to study the transcriptome that governs intra-tumor heterogeneity in HNSCC [80]. These high-resolution strategies will help in delineating the complex tumor–immune interaction occurring within the tumor immune microenvironment [81]. This ultimately allows for the stratification of distinct cellular subpopulations within a tumor in order to better define the immune status of a tumor. 

Another strategy to consider in studying and stratifying tumors is DNA and histone methylation analysis. Antigen Processing Machinery (APM) components and IFN-γ stimulated genes (ISG) are transcriptionally silenced in tumors through epigenetic silencing mediated by DNA or histone methylation [82,83]. Therefore, using DNA and histone methylation analysis to identify the transcriptional state of these APM and ISG genes will also help in predicting patient’s response to immunotherapy, with the transcriptional status of these genes defining tumor immune status as being either HOT or COLD. Epigenomic diagnostics have shown DNA methylation of various prognostic transcription factors and immune checkpoints (PITX2, SHOX2, SEPT9, PD-1, and CTLA4) to be correlated with HNSCC and potentially associated with targeted therapies [84]. Clinical assays should be developed to monitor the DNA methylation status utilizing patient DNA. Depending on the epigenetic state, DNMT inhibitors or the recently FDA-approved drug, tazemetostat could be better utilized in the clinic in combination with immunotherapy and increase overall survival (Figure 1A) [85].

Additionally, various studies of independent prognostic-related proteins (IPP) and IPP signatures are being conducted in order to identify proteomic signatures that can function as prognostic markers that guide management and provide targeted therapy options [86,87]. More recently, YAP1, a downstream transcriptional component of the Hippo pathway, has been shown to contribute to resistance mechanisms in HNSCC [88]. FAT1, the upstream negative regulator of YAP1 is mutated in 30% of HNSCC. Normally, FAT1 through a cascade of signaling events phosphorylates YAP1, thereby retaining it in the cytoplasm and inhibiting the oncogenic transcriptional program [89]. FAT1 mutation or phosphorylation of YAP1 could be utilized as a biomarker for predicting the outcome, disease recurrence, and response to trametinib in patients (Figure 1B) [90]. Metabolomic markers could be integrated with epigenomic and proteomic markers and could be the focus for the future to develop novel biomarkers in HNSCC. Oncometabolites such as acylcarnitine and 2-hydroxyglutarate have been identified as potentially non-invasive biomarkers; however, studies validating the clinical efficacy of these targets must be conducted to validate these findings and have a clinical application [91,92]. 

Plasma-based diagnostic markers have also grown to become an area of emphasis, as this would function as a virtually non-invasive means of diagnosis or treatment monitoring of HNSCC [93]. Plasma Melanoma-Antigen (MLANA) recognized by T-cells has been identified as an effective plasma-based biomarker in monitoring HNSCC patients undergoing chemoradiation therapy [94]. Investigation of extracellular vesicles, exosomes, containing potential biomarkers has indicated the potential to guide future therapies and establish more sensitive monitoring of existing therapies [95]. These exosomes have been shown to carry PDL1, which is upregulated by IFN-γ and suppresses CD8+ T cell function, and facilitates tumor growth without direct expression on the tumor surface [96].

Various tumor- and immune cell-derived immune factors collectively determine the tumor immune microenvironment (TIME) characteristics. Among several cytokine and cytokine receptors, the colony-stimulating factor-1 (CSF-1) secreted by tumor cells interacts with various immune cells in the environment, particularly tumor-associated macrophages (TAM). Such interaction induces TAM into immune suppressive M2 types promoting immune suppression and tumor progression [97]. A high level of M2 TAM in the tumor microenvironment is associated with poor prognosis in various tumors including HNSCC and also resistance to ICIs [98,99,100,101]. CSF-1/CSF1-targeting investigational agents are being evaluated alone or in combination with ICI [102,103].

Additionally, the use of specialized tissue models that better mimic native tumor microenvironments, including the immune signatures and intratumor heterogeneity, allow for more accurate testing of targeted therapies [104]. A relatively new tissue model that has the potential to revolutionize the way cancers are studied and, in turn, treated, is the organoid model. An organoid model is a 3D, multicellular in vitro tissue construct that mimics a corresponding in vivo organ and can be utilized to study aspects of physiology and pathologies associated with that organ [105]. Organoids have been shown to be able to express the diversity of carcinoma subtypes and are capable of predicting in vivo drug sensitivity better than other models [106]. In the case of HNSCC, organoids have the potential to open the door to entirely new subsets of immune markers for targeted therapy, as drug screens have shown sensitivity to targeted drugs that have not been traditionally used in treating HNSCC (Figure 1C) [107]. In conclusion, the development of potential novel biomarkers and accompanying sensitive clinical diagnostic testing tools is important to design better treatment strategies. This will allow for the development of targeted immunotherapies that alleviate the morbidity and mortality associated with HNSCC.

## Figures and Tables

**Table 1:** PD-L1 biomarker data in HNSCC.

Studies	Treatment	PD-L1 Assay	Positivity Cut-Off	Outcomes	References
KEYNOTE-012 (NCT01848834)	Pembrolizumab	PD-L1 IHC 22C3 PharmDx	TPS ≥ 1 CPS ≥ 1	ORR (*p* = 0.023) PD-L1+ (21%) PD-L1− (6%) OS (*p* = 0.008) PD-L1+ (10 months) PD-L1− (5 months)	[19,20]
KEYNOTE-055 (NCT02255097)	Pembrolizumab	PD-L1 IHC 22C3 PharmDx	CPS ≥ 1 CPS ≥ 50	ORR (CPS ≥ 1) PD-L1+ (18%) PD-L1− (12%) ORR (CPS ≥ 50) PD-L1+ (27%) PD-L1− (13%)	[21,22]
KEYNOTE-040 (NCT02252042)	Pembrolizumab vs. Investigator’s Choice (IC)	PD-L1 IHC 22C3 PharmDx	TPS ≥ 50 CPS ≥ 1	OS Pembro vs. IC (TPS ≥ 50) HR: 0.53, *p *= 0.0014 Pembro vs. IC (CPS ≥ 1) HR: 0.74, *p *= 0.0049	[11,23,24,25]
CHECKMATE-141 (NCT02105636)	Nivolumab vs. Investigator’s Choice (IC)	PD-L1 IHC 28–8 PharmDx	TC ≥ 1	OS 7.7 vs. 3.3 months, HR: 0.56 12-month OS 39.2% vs. 15.4% 24-month OS 20.4% vs. 3.8% ORR 20.0% vs. 11.5%	[26]
KEYNOTE-048 (NCT02358031)	Pembrolizumab vs. Pembrolizumab + chemotherapy vs. Cetuximab + chemotherapy (Extreme)	PD-L1 IHC 22C3 PharmDx	CPS ≥ 1 CPS ≥ 20	OS Pembro vs. Extreme (CPS ≥ 20) 14.9 vs. 10.7 months HR: 0.61, *p *= 0.0007 Pembro vs. Extreme (CPS ≥ 1) 12.3 vs. 10.3 months HR: 0.78, *p *= 0.0086 Pembro+ vs. Extreme (CPS ≥ 20) 14.7 vs. 11.0 months HR: 0.60, *p *= 0.0004 Pembro+ vs. Extreme (CPS ≥ 1) 13.6 vs. 10.4 months HR: 0.65, *p *< 0.0001	[27,28,29]
HAWK	Durvalumab	VENTANA PD-L1 SP263	TC ≥ 25%	ORR Total: 16.2% HPV+: 29.4% HPV−: 10.9% OS mOS: 7.1 months 12-month OS: 33.6%	[30]
CONDOR	Durvalumab vs. tremelimumab vs. durvalumab + tremelimumab	VENTANA PD-L1 SP263	TC ≥ 25%	Durvalumab ORR: 9.2% Tremelimumab ORR: 1.6% Durvalumab + Tremelimumab ORR: 7.8%	[31]
EAGLE	Durvalumab vs. Durvalumab + tremelimumab vs. Investigator’s Choice (IC)	VENTANA PD-L1 SP263	TC ≥ 25%	OS Durvalumab PD-L1+ (9.8 months) PD-L1− (7.6 months) Durvalumab + Tremelimumab PD-L1+ (4.8 months) PD-L1− (7.8 months) IC PD-L1+ (9.0 months) PD-L1− (8.0 months)	[32]
